# Maintained and Delayed Benefits of Executive Function Training and Low-Intensity Aerobic Exercise Over a 3.5-Year Period in Older Adults

**DOI:** 10.3389/fnagi.2022.905886

**Published:** 2022-07-01

**Authors:** Lixia Yang

**Affiliations:** Department of Psychology, Toronto Metropolitan University, Toronto, ON, Canada

**Keywords:** maintained benefits, delayed benefits, executive function training, aerobic exercise, transfer effects, older adults

## Abstract

This is a follow-up study of our previous work, with a specific goal to examine whether older adults are able to maintain or show delayed cognitive and psychosocial benefits of executive function training and physical exercise over a period of 3.5 years on average. Thirty-four participants from the original training study (17 from the executive function training and 17 from the aerobic exercise group) returned and completed a single follow-up session on a set of cognitive and psychosocial outcome measures. The results of the returned follow-up sample showed some significant original training transfer effects in WCST-64 performance but failed to maintain these benefits at the follow-up session. Surprisingly, episodic memory performance showed some significant improvement at the follow-up relative to baseline, signaling delayed benefits. The findings add some novel implications for cognitive training schedule and highlight the possible importance of continuous engagement in long-term cognitive enhancement in healthy older adults.

## Introduction

Aging is associated with progressive declines in physical health, as well as social and cognitive functions (de Magalhães et al., 2017). With the global population rapidly aging, it becomes urgent to identify effective training approaches to promote and maintain psychosocial and cognitive health in later years (Green et al., 2019; Yang et al., 2020). Past research has revealed some promising evidence for cognitive enhancement effects through training or intervention programs (Hertzog et al., 2008; Sprague et al., 2019), including cognitive retest practice, strategy-based training, or life style engagement (Wilkinson and Yang, 2015). Cognitive training studies focus on three main effects (Yang, 2011): practice/training effect (i.e., does training improve cognitive performance?), transfer effect (i.e., does training benefit performance on other tasks?), and durability/maintenance effect (i.e., can the training benefits be maintained for a long period of time?). The current study focuses on maintained and delayed benefits from executive function training in comparison to aerobic physical exercise over a delay of 3.5 years on average among healthy older adults.

Executive functions refer to a group of top-down mental processes that serve external or internal goals, including processes such as inhibition, working memory, and cognitive flexibility (Miyake and Friedman, 2012; Diamond, 2013; Karbach and Kray, 2021). These processes are essential for almost all aspects of daily living such as cognitive performance, physical health, mental functions, and quality of life (Diamond, 2013), but show substantial declines with aging (Grady, 2012). Executive function deficits are assumed to underlie other well-reported cognitive declines in older adults, such as speed or working memory (Lustig et al., 2007). In this context, a growing number of studies aim to examine the neural and cognitive benefits of executive function training (Adnan et al., 2017; Nguyen et al., 2019; Yang et al., 2020) or to enhance executive functions through cognitive training (Chambon and Alescio-Lautier, 2019) or physical exercise (Best et al., 2014; Chen et al., 2020; Xiong et al., 2021) among older adults.

Cognitive training studies typically demonstrate transfer distance effects (Zelinski, 2009; Wilkinson and Yang, 2015), with more benefits on structurally similar tasks or tasks measuring the same abilities (near transfer) than tasks assessing other cognitive abilities (near-far transfer) or other functional domains (far transfer). A recent meta-analysis suggests that training core executive functions hold both immediate and long-term cognitive benefits in older adults (Nguyen et al., 2019). Far transfer effects are absent or rarely seen in some studies (Dahlin et al., 2008; Li et al., 2008; Wilkinson and Yang, 2016a), but are present in other studies with executive function training (Jaeggi et al., 2010; Karbach and Verhaeghen, 2014) or general cognitive stimulation/engagement (Stine-Morrow et al., 2008; Park S. J. et al., 2014). However, These positive far transfer results have been challenged in some re-analyses of the data (Melby-Lervåg and Hulme, 2016). Nevertheless, our recent work established the efficacy of self-guided online executive function training with gaming (i.e., *Lumosity*) in improving cognitive flexibility performance (i.e., the computerized 64-card Wisconsin Card Sorting Test, WCST-64) in comparison to an aerobic physical exercise condition in healthy older adults (Yang et al., 2020). The current study follows up this study to further examine whether the training benefits could be maintained, or whether further delayed benefits could be demonstrated over a period of 3.5 years on average.

Physical exercise, specifically aerobic and mind-body exercise, is also effective in improving executive functions in older adults (Hillman et al., 2008; Smith et al., 2010; Xiong et al., 2021). Even low-intensity physical exercise may improve cognitive health in older adults (Tse et al., 2015). However, our recent work failed to reveal any cognitive benefits from a self-guided low-intensity 10-week aerobic exercise regime (Yang et al., 2020). Nevertheless, both executive gaming and low-intensity aerobic exercise tended to reduce depression and stress levels, signaling potential psychosocial benefits among older adults (Yang et al., 2020). The current follow-up study specifically examines whether these benefits last over a 3.5-year delay.

Past research suggests that older adults are able to maintain cognitive training benefits in memory, reasoning, speed, visual attention, and executive functions, with the window of maintenance ranging from a few months to 10 years (Willis et al., 2006; Li et al., 2008; Yang and Krampe, 2009; Rebok et al., 2014; Ji et al., 2016; Wilkinson and Yang, 2016b). A recent meta-analysis also suggests that executive function training benefits can be maintained over time for up to 18 months (Nguyen et al., 2019). However, these studies mostly used lab-based cognitive practice or training, it is still unclear whether older adults show maintained or delayed benefits of self-guided home-based cognitive gaming or exercise. Given mobility restrictions that older adults may have, self-guided home-based training may be more ecologically friendly and convenient to implement for older adults. This is particularly applicable in situations that require social distancing, such as the current COVID-19 pandemic. Additionally, given the lack of close monitoring of the training sessions, regular adherence to home-based training may heavily rely on self-regulation and intrinsic motivation, which is critical for long-term maintenance or delayed benefits. Therefore, the current study aims to fill this gap to examine maintained and delayed cognitive and psychosocial benefits of executive function and physical training.

Importantly, long-term maintenance and/or delayed benefits of training would signal meaningful performance improvement. While immediate, temporary benefits may be superficial and task-specific, durable benefits across a long delay are likely to occur at a fundamental ability-level, and thus are likely to be applied or generalized to everyday life functions or performance. As such, there is a practical importance to evaluate the long-term durability of training benefits, which is the main objective of the current study.

Taken together, the current study follows a recent training program which examined and compared the cognitive and psychosocial benefits of a web-based executive function training (based on Lumosity games) and a low-intensity aerobic exercise program among healthy older adults (Yang et al., 2020). Specifically, the study aims to examine long-term maintenance of the original benefits and delayed benefits at the follow-up session. In the original study (Yang et al., 2020), older adults were enrolled in a 10-week cognitive training program based on a set of online executive function games^1^ or a low-intensity aerobic exercise program following a set of Digital Video Disc (DVDs) for 25–30 min per day, 4 days a week.

## Materials and Methods

### Participants

The original study (Yang et al., 2020) consisted of 40 healthy older adults (aged 65–87; *M* = 70.83, *SD* = 5.25) recruited and pre-screened from the Ryerson Senior Participant Pool (RSPP). They were evenly and randomly assigned to either an executive function training regime or a low-intensity aerobic exercise program. All participants were invited, and 34 returned (age range at follow-up was 68–90; *M* = 74.50, *SD* = 5.42), for a single 1-h follow-up session at a delay of approximately 3.5-years on average (*M* = 42.86, *SD* = 6.18 in months) from the post-test session of the original study, ranging from 34 to 60 months. Table 1 displays the demographic information and baseline cognitive or physical performance of the returned and not returned participants. At baseline (pretest), all participants completed the following tasks: the Modifiable Activity Questionnaire (MAQ, Yang et al., 2020) assesses physical activity level. It was originally developed and validated by Aaron et al. (1995) to assess physical activity, as indexed by the time engaging in any of the 40 physical activities in the past year. It was modified in the current study to cover a 1-month period in the past year (by identifying the average number of times an activity was engaged and the average length of time in minutes spent on that activity each time); the Cognitive Activity Questionnaire (CAQ, Yang et al., 2020) measures engagement in 12 cognitively stimulating activities based on a 5-point Likert Scale (i.e., 1 = “once a year or less” and 5 = “every day or about every day”). It is a lab-made scale developed in our own lab (not yet validated with a separated sample) and used to provide a parallel assessment of cognitive activity levels; the Home Step Test (Wood, 2010) assesses physical fitness (i.e., stepping up and down on an exercise step for 3 min while heart rate was recorded before and after); and the Mini-Mental State Examination (MMSE, Folstein et al., 1975) as a screen for potential dementia-related cognitive impairments. The drop-out participants scored lower on the MMSE than those returned in the executive function training group (*p* = 0.004), presumably because the cognitive demand in the executive function training program might have discouraged those with declined general cognitive functioning from continued participation in this follow-up session. No other attrition bias was found in both training groups (*p*s ≥ 0.071). Participants were compensated $15 for their time and debriefed at the end of the session.

**TABLE 1:** Baseline demographic information of those who returned for the follow-up vs. those who did not (i.e., not returned) across the executive function and physical training groups.

	Executive function training			Physical training		
	Returned (*n* = 17)	Not Returned (*n* = 3)	*P*	Returned (*n* = 17)	Not Returned (*n* = 3)	*p*
Age (years)	72.65 (6.34)	70 (3.00)	0.495	69.12 (3.82)	71.00 (6.00)	0.475
Gender (male:female)	3:14	2:1	0.071	3:14	2:1	0.071
Formal education (years)	18.38 (2.68)	18.00 (1.73)	0.821	16.94 (2.19)	17.33 (5.51)	0.823
Health rating	8.29 (0.92)	8.67 (1.15)	0.530	8.76 (1.15)	8.67 (1.53)	0.897
MAQ (minutes)	25,684.00 (20,811.55)	19,040.00 (10,667.41)	0.601	28,506.82 (19,643.00)	49,036.00 (33,551.00)	0.147
CAQ	3.25 (0.62)	3.31 (0.60)	0.898	3.51 (0.40)	3.94 (0.54)	0.112
MMSE	28.82 (1.13)	26.00 (2.65)	0.004**	28.71 (1.49)	27.00 (1.00)	0.075
Home Step Test (number of steps)	53.86 (8.67)	56.67 (13.32)	0.535	62.40 (13.66)	54.00 (14.00)	0.347
Home Step Test (heart rate increase)	46.67 (17.67)	50.67 (33.50)	0.760	51.38 (17.20)	44.67 (24.03)	0.564
Follow-up delay (months)	43.21 (6.73)			42.51 (5.76)		

Most cells present the mean (M), with Standard Deviation (SD) in parenthesis, except for the gender cells which present a ratio score. Healthy rating was based on a Likert scale of 1 (least healthy) to 10 (monst healthy). Between-group one-way ANOVA was run for all variables except for gender ratio, which was analyzed with a Pearson’s Chi square test. MAQ, the Modifiable Activity Questionnaire; CAQ, the Cognitive Activity Questionnaire; MMSE, the Mini-Mental State Examination. Heart rate was assessed in beats per minute (BPM) and the heart rate increase was calculated by subtracting the baseline BPM from the BPM right after the Home Step Test. P-values refers to the group effect from the one-way ANOVA for each training condition. **p < 0.01. Bold values denote statistically significant effects.

### Procedure and Materials

#### The Original Study Training Protocol

The original study (Yang et al., 2020) adopted a 2 (training: executive function vs. physical) × 2 (session: pretest vs. post-test) mixed-model design, with training program as a between-subjects and session as a within-subjects variable following a standard pretest-training-post-test protocol. A comprehensive battery of cognitive and psychosocial outcome measures was administered at the pretest and post-test sessions within a 1-week window from the first and the last training session respectively. The training used a short-term adaptive training schedule (i.e., 25–30 min per day, 4 days a week for 10 weeks) involving 10 Lumosity games for the executive function training group, and an indoor low-intensity aerobic exercise workout following a set of DVDs (details can be found in Yang et al., 2020) for the physical exercise group. Participants in both conditions completed a training log for each training session. Training completion and adherence rates were high (i.e., 93.88% completed sessions on average), as determined through periodic reminder phone calls, Lumos Labs’ data recordings, heart rate recordings, and the self-reported training log (Yang et al., 2020).

#### Outcome Measures

The comprehensive outcome measures in the original study (Yang et al., 2020) include cognitive near transfer tasks (i.e., N-Back, Stroop, Navon Local-Global, WCST-64, Change Detection), cognitive far transfer tasks [i.e., the Digit Symbol Substitution Test (DSST), the Hopkins Verbal Learning Test-Revised (HVLT-R)], and psychosocial far transfer tasks [i.e., the Depression Anxiety Stress Scales (DASS-21), the Instrumental Activities of Daily Living (IADL)] (see Yang et al., 2020 for detailed description and original sources of these tasks). For the follow-up session, we selected tasks at each transfer level (see below for task descriptions of the selected tasks), taking into consideration reported transfer effects, potential delayed benefits, task length, and participants’ anecdotally reported challenges in the original study. The final package took approximately 1 h to complete. This is a typical and reasonable length in cognitive aging research that allows older adults to attend and complete the tasks with little effect of fatigue or fading interest.

##### Cognitive Near Transfer Tasks

The WCST-64 (Kong et al., 2000) assesses general cognitive control or cognitive flexibility. The dependent performance variables include: (1) *total correct* (i.e., the number of correct trials); (2) *perseverative responses* (i.e., number of cards continuously sorted based on a specific rule, regardless of accuracy); (3) *perseverative errors* (i.e., the number of cards continuously incorrectly sorted based on a previous rule after a rule change); (4) *non-perseverative errors* (i.e., other types of errors); (5) *conceptual level responses* (i.e., instances of three or more consecutive correct responses); (6) *categories completed* (i.e., instances of 10 consecutive correct responses); (7) *trials to complete the first category* (i.e., number of trials needed to successfully complete the first category); (8) *failure to maintain set* (i.e., number of failures to continuously respond based on a correct sorting rule); and (9) *learning to learn* (i.e., change in errors between successive categories). Two participants did not participate in the WCST-64 test, thus the results on WCST-64 were based on 32 participants. The digit N-Back task (Wilkinson and Yang, 2016a) requires participants to identify *via* key pressing whether the currently presented digit is identical to a pre-specified target digit (0-back), the digit presented on the trial right before (1-back), or two trials before (2-back) in three separate blocks. The performance is indexed by hit rate (i.e., the proportion of targets correctly identified) and false alarm (FA) rate (i.e., the proportion of non-targets misidentified as targets) within each block (Yang et al., 2020). The Stroop task (Stroop, 1935; Wilkinson and Yang, 2016b) requires key-pressing responses to identify the ink color in which a color word is printed. It includes three trial types: Congruent (e.g., the word “BLUE” printed in blue ink), incongruent (e.g., the word “BLUE” printed in green ink), and neutral (e.g., “XXXX” printed in blue ink). The dependent variable was the Stroop interference ratio score of incongruent over neutral trials in both RT and accuracy (i.e., RT interference ratio score = RT_incongruent_/RT_neutral_; Accuracy interference ratio score = Hit_incongruent_/Hit_neutral_).

##### Cognitive Far Transfer Tasks

The DSST (Wechsler, 1981) is a processing speed task requiring participants to substitute as many digits as possible with the corresponding symbols based on a given digit-symbol mapping key. The dependent variable is the number of correct substitutions within 2 min. The HVLT-R (Brandt and Benedict, 2020) requires participants to learn and retrieve 12 nouns from 3 semantic categories. Memory is tested by three trials of immediate recall (Trials 1–3), a 20-min delayed recall (Trial 4), and a delayed yes/no recognition test including 12 lures (Trial 5). It results in five dependent variables: (1) *Total immediate recall* across Trials 1–3; (2) *Immediate recall learning slope* (average gains per trial across Trials 1–3); (3) *Delayed recall* (recall at Trial 4); (4) *Retention* (Trial 4 divided by Trial 2 or 3, whichever was higher); and (5) *Recognition discrimination* (hits minus false alarms on Trial 5).

##### Psychosocial Far Transfer Tasks

The DASS-21 (Lovibond and Lovibond, 1995) assesses depression, anxiety and stress levels during the past week based on a 4-point Likert scale from 0 (“did not apply to me at all”) to 3 (“applied to me very much or most of the time”). It has three 7-item subscales, each indexed by the sum score multiplied by 2. The IADL (Lawton and Brody, 1969) assesses functioning in eight daily living activities by selecting an option from the list that most closely describes their current functioning level. Each item was scored “1” only if the activity could be performed with at least a minimal level of functioning. The sum score is used as the dependent variable.

Parallel versions of the outcome measures, wherever possible [i.e., N-Back, Stroop, DSST, and HVLT-R; see Yang et al. (2020)], were administered at the pretest and the post-test sessions, with the order of the two versions counterbalanced across participants. At the follow-up session, we used the same version as the baseline pretest session.

#### General Procedure

At the follow-up session, we first collected signed informed consent from the participants and then administered the aforementioned outcome measures in the following order: N-Back and Stroop (the order of these two tasks counterbalanced across participants), HVLT-R immediate recalls, WCST-64, DSST, IADL, HVLT-R delayed recall and recognition, and DASS-21. Finally, participants completed other tasks in the following order: Home Step Test, MAQ, CAQ, and MMSE. Participants were then debriefed, paid and dismissed.

### Statistical Analysis

The data were analyzed in IBM SPSS version 26. Greenhouse-Geisser criterion was applied if the assumption of sphericity was violated in the Mauchly’s Test. For the Stroop task, only RTs for correct responses were included and RTs were trimmed by removing any RTs that were more than 2.5 standard deviations (SDs) away from the mean for each condition. Given the goal of assessing long-term maintenance or delayed benefits, we reported both the transfer effects and the delayed (at the follow-up session) vs. immediate benefit (at the post-test session) of the follow-up sample. The delayed benefit would signal a long-term maintenance effect of the original immediate training benefit only if the original transfer effect was significant.

## Results

The results were reported in two sections. The first section reported the transfer effects of the follow-up sample. This was done to verify the reported transfer effects in the returned follow-up sample (*n* = 17 for each training group), which would provide a foundation for the examination of the benefit maintenance at the follow-up session. The second section reported the maintenance of the transfer effect (if any) and delayed benefit in comparison to the original immediate benefit for each outcome variable.

### Transfer Effect

Following Yang et al. (2020), the transfer effects were indexed by the within-between interaction in a set of mixed-model 2 (session: pretest vs. post-test) × 2 (group: executive function vs. physical) Analysis of Variance (ANOVA) models, one for each outcome variable. Table 2 presents the performance across pretest, post-test, and the follow-up sessions as well as the original transfer effects (i.e., group by session interaction) of the follow-up sample. The results revealed significant transfer effects, with a larger benefit in the executive function than the physical training group, in a few WCST-64 variables, including total correct, perseverative responses, perseverative errors, non-perseverative errors, conceptual level responses, and trials to complete the first category (*F*s ≥ 4.92; *p*s ≤ 0.034). The transfer effects in other WCST-64 variables or other outcome measure dependent variables were not significant (*p*s ≥ 0.072).

**TABLE 2:** Performance at pretest, posttest, and follow-up sessions, as well as the transfer effects of the follow-up sample (*N* = 34).

Measures	Executive function training			Physical training			Transfer effect	
	Pretest	Posttest	Follow-up	Pretest	Posttest	Follow-up	*p*	η_p_^2^
**Cognitive Near transfer: N-Back**								
0-back hit	0.98 (0.04)	0.99 (0.04)	0.95 (0.11)	0.93 (0.21)	1.00 (0.02)	0.93 (0.24)	0.237	0.043
1-back hit^s^	0.88 (0.13)	0.97 (0.05)	0.91 (0.15)	0.90 (0.12)	0.93 (0.08)	0.92 (0.10)	0.287	0.035
2-back hit	0.75 (0.14)	0.81 (0.11)	0.75 (0.19)	0.85 (0.13)	0.84 (0.10)	0.80 (0.17)	0.127	0.071
0-back false alarm	0.01 (0.03)	0.01 (0.02)	0.01 (0.02)	0.02 (0.05)	0.00 (0.01)	0.00 (0.02)	0.337	0.029
1-back false alarm^s^	0.02 (0.03)	0.02 (0.02)	0.04 (0.11)	0.04 (0.05)	0.02 (0.04)	0.01 (0.02)	0.431	0.019
2-back false alarm	0.08 (0.04)	0.09 (0.05)	0.10 (0.11)	0.07 (0.05)	0.06 (0.04)	0.08 (0.05)	0.389	0.023
**Stroop**								
Accuracy interference^g^	0.90 (0.23)	0.98 (0.05)	0.97 (0.07)	0.99 (0.02)	1.00 (0.02)	0.98 (0.05)	0.135	0.069
RT interference (ms)	1.27 (0.13)	1.27 (0.19)	1.25 (0.16)	1.20 (0.11)	1.21 (0.12)	1.23 (0.12)	0.727	0.004
**WCST-64**								
Total correct^gs^	40.81 (10.72)	47.31 (9.29)	43.13 (11.19)	49.00 (6.15)	47.44 (8.98)	48.38 (8.59)	0.006**	0.222
Perseverative responses^gs^	13.06 (8.65)	9.63 (7.86)	11.81 (8.75)	7.56 (3.52)	9.13 (4.83)	7.75 (4.88)	0.032*	0.145
Perseverative errors^gs^	11.56 (5.57)	8.63 (6.02)	10.88 (7.40)	7.00 (3.03)	8.06 (3.99)	7.06 (3.96)	0.034*	0.141
Non-perseverative errors^gs^	11.63 (5.57)	8.06 (3.84)	10.00 (5.24)	8.00 (3.83)	8.50 (5.55)	8.56 (5.62)	0.030*	0.147
Conceptual level responses^gs^	33.75 (15.26)	42.50 (13.22)	36.94 (16.16)	45.38 (8.09)	42.88 (13.17)	44.31 (12.26)	0.012*	0.194
Categories completed	2.25 (1.61)	2.88 (1.50)	3.00 (1.67)	3.31 (1.35)	3.06 (1.65)	3.38 (1.59)	0.072	0.104
Trials to complete first category^gs^	26.75 (20.00)	16.94 (13.68)	17.63 (13.73)	16.56 (6.78)	21.44 (18.59)	14.00 (6.77)	0.032*	0.145
Failure to maintain set	0.37 (0.72)	0.69 (1.20)	0.25 (0.58)	0.63 (0.96)	0.44 (0.63)	0.44 (0.81)	0.206	0.053
Learning to learn	1.82 (8.41)	−4.35 (6.82)	−1.68 (5.42)	0.85 (7.30)	−1.50 (9.87)	0.55 (4.03)	0.902	0.001
**Cognitive Far Transfer**								
DSST: # Correct Solutions^g^	59.18 (14.06)	59.47 (14.95)	57.35 (11.05)	71.47 (17.07)	73.47 (15.94)	69.65 (16.83)	0.402	0.022
HVLT-R: Immediate recall	25.06 (4.68)	26.53 (5.91)	27.65 (4.90)	26.24 (4.52)	27.12 (4.41)	28.65 (3.52)	0.736	0.004
HVLT-R: Recall learning slope	1.56 (0.81)	1.50 (0.95)	1.62 (0.82)	1.65 (0.81)	1.47 (0.84)	1.59 (0.73)	0.756	0.003
HVLT-R: Delayed recall	8.41 (2.29)	9.29 (2.14)	9.71 (1.69)	9.18 (1.55)	9.71 (1.79)	10.06 (1.64)	0.651	0.006
HVLT-R: Retention	0.84 (0.16)	0.90 (0.13)	0.90 (0.09)	0.90 (0.12)	0.93 (0.11)	0.92 (0.14)	0.614	0.008
HVLT-R: Recognition discrimination^g^	11.00 (1.17)	11.24 (1.03)	11.24 (0.90)	11.47 (0.62)	11.47 (0.62)	11.44 (0.63)	0.576	0.010
**Psychosocial Far Transfer:**								
DASS-21: Depression^g, s^	6.00 (8.15)	3.06 (5.88)	4.82 (3.88)	1.76 (1.86)	0.94 (2.56)	1.65 (2.94)	0.153	0.063
DASS-21: Anxiety	4.47 (4.87)	4.71 (5.38)	7.29 (4.41)	1.29 (2.11)	0.65 (1.17)	1.88 (2.39)	0.508	0.014
DASS-21: Stress^g, s^	10.47 (5.64)	8.82 (4.90)	8.94 (4.31)	5.18 (5.15)	2.59 (3.59)	3.06 (3.01)	0.544	0.012
IADL: Sum score	7.88 (0.33)	7.88 (0.33)	7.76 (0.56)	7.71 (0.77)	7.88 (0.33)	7.94 (0.24)	0.178	0.056

*WCST-64, 64-card Wisconsin Card Sorting Task; DSST, Digit Symbol Substitution Task; HVLT-R, Hopkins Verbal Learning Test-Revised; DASS-21, 21-item Depression Anxiety Stress Scales; IADL, Instrumental Activities of Daily Living Scale.*

*p and η_p_^2^ values are for the Group × Session interaction that signals the transfer effects.*

*^g^Group effect was significant.*

*^s^Session effect was significant.*

*^gs^Group × Session interaction was significant.*

*p < 0.05 and **p < 0.01.

*Bold values denote statistically significant effects.*

### Delayed vs. Immediate Benefit

Following previous practice (Wilkinson and Yang, 2020), we calculated the effect size scores for the immediate benefit at the post-test session (i.e., dividing the pre-post performance difference by pretest SD) and the delayed benefit at the follow-up session (i.e., dividing the follow-up vs. pretest performance difference by pretest SD) of the follow-up sample. Effect size scores allow a standardized comparison across tasks and sessions. The effect size scores were submitted to a set of mixed-model 2 (session: post-test vs. follow-up) × 2 (group: executive function vs. physical) ANOVAs. Significant effects were followed up with the imbedded pairwise comparisons with Bonferroni correction. Additionally, we ran planned one-sample *t-*tests to evaluate whether these effect size scores were significant for each group at each session, given our hypothesis-driven interest in the group-specific training benefits (i.e., whether the effects were significant in executive function but not physical training group). Table 3 displays the immediate and delayed benefit effect size scores, and the corresponding *p-*values.

**TABLE 3:** Immediate and delayed benefit effect size in the unit of baseline SD (*N* = 34).

Measures	Executive function training				Physical training				Interaction
	Immediate Benefit	*p*	Delayed Benefit	*p*	Immediate Benefit	*p*	Delayed Benefit	*p*	*P*
**Cognitive Near transfer: N-Back**									
0-back hit	0.03 (0.42)	0.750	−0.18 (0.67)	0.294	0.46 (1.41)	0.194	0.11 (2.23)	0.984	0.585
1-back hit	0.71 (1.14)	0.021*	0.23 (1.48)	0.525	0.32 (0.97)	0.200	0.16 (0.74)	0.385	0.423
2-back hit	0.41 (1.12)	0.153	−0.23 (1.15)	0.935	−0.13 (0.87)	0.543	−0.36 (1.22)	0.244	0.615
0-back false alarm	−0.13 (0.49)	0.299	−0.06 (0.47)	0.616	−0.43 (1.10)	0.154	−0.41 (1.38)	0.235	0.757
1-back false alarm	−0.06 (0.74)	0.753	0.58 (2.73)	0.393	−0.32 (1.16)	0.269	−0.56 (1.32)	0.101	0.227
2-back false alarm	0.18 (0.87)	0.418	0.48 (2.25)	0.390	−0.13 (1.18)	0.644	0.25 (1.18)	0.493	0.903
**Stroop**									
Accuracy interference	0.47 (1.09)	0.095	0.43 (1.06)	0.114	0.06 (0.11)	0.034*	−0.04 (0.32)	0.619	0.480
RT interference (ms)	−0.01 (1.46)	0.986	−0.13 (0.97)	0.577	0.13 (0.63)	0.410	0.26 (0.96)	0.288	0.571
**WCST-64**									
Total correct^g,gs^	0.68 (0.83)	0.005**	0.24 (0.79)	0.237	−0.16 (0.80)	0.424	−0.07 (0.57)	0.653	0.030*
Perseverative responses^g^	−0.49 (0.96)	0.060	−0.18 (0.53)	0.200	0.22 (0.81)	0.293	0.03 (0.53)	0.845	0.106
Perseverative errors	−0.48 (0.94)	0.056	−0.11 (0.53)	0.397	0.18 (0.73)	0.353	0.01 (0.47)	0.931	0.057
Non-perseverative errors	−0.71 (1.19)	0.031*	−0.32 (1.15)	0.282	0.10 (0.77)	0.612	0.11 (0.73)	0.553	0.223
Conceptual level responses^g^	0.65 (0.90)	0.011*	0.24 (0.90)	0.307	−0.19 (0.87)	0.402	−0.08 (0.60)	0.606	0.056
Categories completed	0.40 (0.81)	0.066	0.48 (1.03)	0.083	−0.16 (0.89)	0.483	0.04 (0.98)	0.872	0.761
Trials to complete first category	−0.63 (1.10)	0.037*	−0.56 (1.22)	0.075	0.31 (1.26)	0.337	−0.16 (0.65)	0.327	0.090
Failure to maintain set	0.37 (1.20)	0.237	−0.15 (0.59)	0.333	−0.22 (1.39)	0.530	−0.22 (1.52)	0.566	0.325
Learning to learn	−0.27 (1.34)	0.646	−0.61 (1.61)	0.351	−0.36 (1.48)	0.439	−0.65 (1.35)	0.883	0.557
**Cognitive Far Transfer**									
DSST: # Correct Solutions^s^	59.18 (14.06)	0.886	57.35 (11.05)	0.199	71.47 (17.07)	0.198	69.65 (16.83)	0.366	0.637
HVLT-R: Immediate recall	0.33 (1.29)	0.322	0.44 (0.77)	0.036*	0.19 (0.98)	0.444	0.58 (0.80)	0.012*	0.486
HVLT-R: Recall learning slope	−0.03 (1.42)	0.915	0.04 (1.42)	0.915	−0.35 (1.26)	0.286	−0.16 (1.19)	0.609	0.806
HVLT-R: Delayed recall	0.50 (1.26)	0.135	0.53 (0.71)	0.010*	0.26 (0.99)	0.285	0.44 (0.88)	0.056	0.676
HVLT-R: Retention	0.49 (1.25)	0.137	0.39 (1.09)	0.163	0.26 (0.97)	0.291	0.18 (1.44)	0.622	0.996
HVLT-R: Recognition discrimination	0.24 (1.60)	0.543	0.24 (1.33)	0.466	0.06 (0.70)	0.718	0.00 (0.67)	1.000	1.000
**Psychosocial Far Transfer:**									
DASS-21: Depression	6.00 (8.15)	0.053	4.82 (3.88)	0.620	1.76 (1.86)	**0.034**	1.65 (2.94)	0.708	0.510
DASS-21: Anxiety^s^	4.47 (4.87)	0.922	7.29 (4.41)	0.060	1.29 (2.11)	0.186	1.88 (2.39)	0.580	0.184
DASS-21: Stress	10.47 (5.64)	0.252	8.94 (4.31)	0.476	5.18 (5.15)	**0.010**	3.06 (3.01)	0.144	0.939
IADL: Sum score	7.88 (0.33)	—-	7.76 (0.56)	0.580	7.71 (0.77)	0.188	7.94 (0.24)	0.216	0.332

*WCST-64, 64-card Wisconsin Card Sorting Task; DSST, Digit Symbol Substitution Task; HVLT-R, Hopkins Verbal Learning Test-Revised; DASS-21, 21-item Depression Anxiety Stress Scales; IADL, Instrumental Activities of Daily Living Scale.*

*p values in the first four columns are for the one-sample t-tests and p values in the final column are for the Group by Session interaction that signals maintained benefits.*

*^g^Group effect was significant.*

*^s^Session effect was significant.*

*^gs^Group × Session interaction was significant.*

*p < 0.05 and **p < 0.01.

*Bold values denote statistically significant effects.*

**FIGURE 1:**
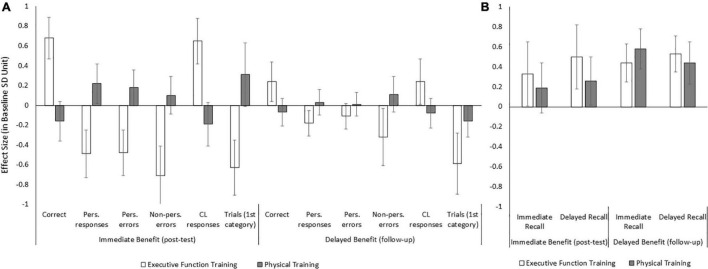
The effect size scores of immediate benefit at the post-test session and delayed benefit at the follow-up session in WCST-64 **(A)** and HVLT-R **(B)** outcome variables for the executive function and physical training groups..

The overall 2 × 2 ANOVA revealed a significant session effect in DSST (*p* = 0.030) and DASS-21 anxiety (*p* = 0.001), a group effect in WCST-64 total correct (*p* = 0.022), perseverative responses (*p* = 0.038), conceptual level responses (*p* = 0.035), and a group by session interaction in WCST-64 total correct (*p* = 0.030). All the other effects were not significant (*p*s ≥ 0.056). The imbedded pairwise comparisons for the group by session interaction in WCST-64 total correct showed a session effect in the executive function training group, with a larger immediate than delayed benefit (*p* = 0.013), but not in the physical training group (*p* = 0.559). Based on the planned one-sample *T*-test analyses (see Table 3), the immediate benefit effect size was significant in 1-back hit and a few WCST-64 variables for the executive function training group (*p*s ≤ 0.037), Stroop accuracy interference, and DASS-21 depression and stress scores for the physical training group (*p*s ≤ 0.034). The delayed benefit effect size was significant in HVLT-R immediate and delay recall (*p*s ≤ 0.036) for the executive function training group. It was significant in HVLT-R immediate recall (*p* = 0.012) and approaching significance in delayed recall (*p* = 0.056) for the physical training group. Figure 1 illustrates these significant immediate and delayed benefit effect sizes in WCST-64 and HVLT-R for each group. Given the lack of the immediate benefit effects in the HVLT-R, we argue that the delayed benefit at the follow-up session here largely reflects a delayed practice effect instead of training maintenance.

No significant correlations were found between the demographic variables (i.e., age, education, health rating, and baseline MMSE) and the critical outcome variables (i.e., the immediate benefits of the six WCST-64 outcome variables and the delayed benefits in HVLT-R immediate and delayed recall), *r*s ≤ 0.320, *p*s ≥ 0.069. The two gender groups did not differ in all these outcome variables (*p*s ≥ 0.246). Thus demographic variables were not included as covariates in the final data analysis.

## Discussion

The current study is a follow-up to our previous work (Yang et al., 2020) to explore the long-term maintenance and delayed benefits of a self-guided home-based online executive function training program and a self-guided home-based physical aerobic exercise program over a 3.5-year period on average in healthy older adults. Overall, the original executive function training resulted in some significant near transfer effects in performance on some WCST-64 outcome variables, but the immediate benefits at the post-test session on any of these variables did not last over a 3.5-year delay, suggesting the lack of a maintenance effect of the original training transfer effects. The physical training group showed significant immediate benefits in Stroop accuracy interference and DASS-21 depression and stress scores, but none of these variables showed delayed benefits at the 3.5-year follow-up. Surprisingly, memory performance (HVLT-R immediate and delayed recall) showed a delayed benefit at the 3.5-year follow-up compared to the baseline performance at pretest, in both training groups. This may reflect a memory practice effect that occurs over a longer delay.

Taken together, the results suggest that, although the self-guided home-based online executive function game training resulted in immediate near transfer effects to a general executive control task (i.e., WCST-64), these benefits faded away after a 3.5-year delay. These results suggest that the training benefits were not durable for a 3.5-year-long period. The results were inconsistent with other studies which showed long-term maintenance of a computerized multi-task executive function (i.e., inhibition specifically) training program over a period of 3.5-years (Wilkinson and Yang, 2020). It should be noted that the training in Wilkinson and Yang (2020) involved extensive practice of critical computerized inhibition tasks (e.g., letter N-Back), which might have sharpened the implicit procedural performance learning that relied on the same inhibition skills as required in the structurally similar inhibition transfer tasks (e.g., digit N-Back). The near transfer task (i.e., WCST) in the current study does not overlap with any training games in task structure, and thus the immediate transfer effect in these tasks likely occurred at a more conceptual level (requiring the similar task rules, for example), which might not be sustainable over a long period of time. However, this speculation needs to be verified in future studies. Furthermore, the original psychosocial benefits in depression and stress (especially in the physical training group) also did not last over the 3.5-year delay. This further suggests that the original reduction in depression and stress might be due to the gradually decreased stress or excitement overall with habituation and familiarization of the same tasks or testing situations over time (Yang et al., 2020). Nevertheless, the lack of the long-term maintenance effect in the current study urges us to further explore the critical factors or mechanisms for the durability of cognitive training benefits. Together with previous findings (Wilkinson and Yang, 2020), we speculated that similarity/overlap in task structure and underlying perceptual processes between the training and transfer tasks might be key for the long-term maintenance of the training benefits. This may somewhat support the theory of transfer-appropriate processing (TAP, Morris et al., 1977) which assumes that optimum memory performance occurs when the same processes are engaged across encoding and retrieval. For example, past research revealed largest repetition priming when exactly the same tasks were given across the two testing phases (Franks et al., 2000).

Surprisingly, the results showed some long-term delayed improvement in episodic memory performance in HVLT-R immediate and delayed recall despite the lack of immediate training benefits. We speculate that this delayed benefit might be driven by the continued engagement of similar physical or cognitive activities that they learned/acquired during the original 10-week training, over the delay period. Furthermore, exposure to the original training might have motivated and elicited a positive attitude toward new learning and/or promoted an active and engaging lifestyle that could have in turn enhanced overall memory performance. In support of this assumption, previous work did show that a 14-week productive engagement (e.g., learning new skills such as quilting or photography) program (i.e., the Synapse Project) significantly enhanced episodic memory performance (Park D. C. et al., 2014). Furthermore, the supplementary analysis on the MAQ and CAQ performance across the pretest to the follow-up session by the two training groups showed no differences between the two training groups (*p*s ≥ 0.144), nor across the two sessions (*p*s ≥ 0.669). The group by session interaction was also not significant (*p*s ≥ 0.742). These results support the continuous engagement of some general physical and cognitive activities over the 3.5-year delay in both training groups. Of note, participants were not asked to continue with the exercise or gaming practice during the delay and they were also not informed about this follow-up session ahead of time. Thus the long-term delayed memory boost is quite impressive and inspiring. However, we acknowledge that this explanation is still speculative considering that we did not track the frequency or duration of continued cognitive/physical engagement during the delay and we did not have a no-training control group to serve as a comparison baseline. Nevertheless, this inspiring result and speculation warrants further exploration in the future.

The current study also has some limitations. The sample size was small, but comparable to other long-term cognitive training maintenance studies (Wilkinson and Yang, 2020). As well, the delay interval window varied according to individual participants’ availability, making it hard to draw a firm conclusion about the actual length of the delay. Furthermore, the physical exercise was low-intensity and may have restricted the possibility of training benefits. Additionally, the lack of the no-training control and engagement tracking during the delay greatly restricted the interpretations for the significant delayed benefit effects on memory performance (e.g., observed in the HVLT-R). Finally, we are unable to completely rule out the retest practice effects, but we do not think it is the primary contributor based on the following considerations: (1) we administered parallel versions of some cognitive outcome measures wherever possible [i.e., N-Back, Stroop, DSST, and HVLT-R; see details in Yang et al. (2020)], to minimize the item-specific practice effect in the original transfer effect; (2) the transfer effect was indexed by the group by session interaction to assess whether the pre- to post- improvement (if any) was differentially larger in the executive function training than the physical training condition, thus the retest practice effect would have been well controlled for, and; (3) the delayed benefit effect only occurred in HVLT-R but not in all the other tasks, despite having used the same version as the baseline pretest version across all the outcome measures.

Nevertheless, the current study added some novel contributions to the literature by examining the long-term maintenance and delayed benefits of a self-guided, home-based, gaming-specific cognitive training and a low-intensity aerobic physical training program in healthy older adults. The lack of the durability/maintenance effect in the current study urges us to further examine the factors or mechanisms for cognitive training maintenance. Consistent with the TAP (Morris et al., 1977), it is speculated that the structural and perceptual process overlap between training and transfer tasks might be key for long-term benefit maintenance. The results also shed some light on the delayed memory boost effect likely driven from continuous engagement with an active lifestyle as elicited by an engagement-oriented training program. Future studies may follow up to further examine this potential for cognitive benefit maintenance and delayed memory benefits with highly engaging cognitive or physical stimulation programs.

## Author’s Note

The original practice and transfer effects of the full sample of 40 participants have been presented at the Canadian Psychological Association (CPA) Annual Convention (2017) and the Rotman Research Institute Conference (2019) and published in *Frontiers in Aging Neuroscience* (Yang et al., 2020). However, the data reported in this manuscript, including the original training gains and delayed benefits at the follow-up session of the returned follow-up sample (*n* = 17 in each group) have never been disseminated or reported elsewhere.

## Data Availability Statement

The SPSS data, syntax, and output files could be retrieved from https://doi.org/10.17605/OSF.IO/MNECB.

## Ethics Statement

This study received the ethics approval from the Research Ethics Board (REB) of Ryerson University (currently as the Toronto Metropolitan University). The patients/participants provided their written informed consent to participate in this study.

## Author Contributions

The author contributed to the design, data analysis, and manuscript preparation.

## Conflict of Interest

The author declares that the research was conducted in the absence of any commercial or financial relationships that could be construed as a potential conflict of interest.

## Publisher’s Note

All claims expressed in this article are solely those of the authors and do not necessarily represent those of their affiliated organizations, or those of the publisher, the editors and the reviewers. Any product that may be evaluated in this article, or claim that may be made by its manufacturer, is not guaranteed or endorsed by the publisher.

## Footnotes

1
http://www.lumosity.com
